# Occurrence and Characterization of *Salmonella* Isolated From Chicken Breeder Flocks in Nine Chinese Provinces

**DOI:** 10.3389/fvets.2020.00479

**Published:** 2020-08-05

**Authors:** Yan Song, Fangkun Wang, Yang Liu, Yanying Song, Lin Zhang, Fuyou Zhang, Xiaoxue Gu, Shuhong Sun

**Affiliations:** ^1^Department of Preventive Veterinary Medicine, College of Veterinary Medicine, Shandong Agricultural University, Taian, China; ^2^Shandong Provincial Key Laboratory of Animal Biotechnology and Disease Control and Prevention, Shandong Agricultural University, Taian, China; ^3^China Animal Disease Control Center, Beijing, China

**Keywords:** *Salmonella*, multidrug resistance, MLST, phenotypes, chickens, breeder farms

## Abstract

We investigated the prevalence of salmonellosis on 17 poultry breeding farms in nine Chinese provinces (Shandong, Jiangsu, Anhui, Zhejiang, Fujian, Guangdong, Yunnan, Sichuan, and Chongqing). Altogether, 3,508 samples from poultry breeding farms were collected in 2019, including 1,400 from cloaca swabs, 210 from feed, 1,688 from chicken embryos, and 210 from water. All the samples were subjected to bacterial isolation and culture, and bacterial species were identified by polymerase chain reaction. Serotyping, multilocus sequence typing (MLST), and drug-resistance phenotyping were performed on the isolates identified as *Salmonella*. Altogether, 126 *Salmonella* strains were detected in the 3,508 samples and the positivity rate for the samples was 3.59%. Among all the strains, 95 Salmonella isolates were selected for antimicrobial susceptibility test, resistance gene detection, serotyping, and genotyping. *S. gallinarum-pullorum* (57/95, 60.00%), *S. enteritidis* (22/95, 23.16%), and *S. agona* (16/95, 16.84%) serotypes were identified. The MLST classification showed that the 95 *Salmonella* strains fell into the following five sequence types (STs): ST92 (37/95, 38.95%), ST11 (22/95, 23.16%), ST2151 (19/95, 20.00%), ST13 (16/95, 16.84%), and ST470 (1/95, 1.05%). Apart from ST13, the other four STs shared close genetic relationships, and the genetic direction was ST11-ST470-ST92-ST2151. The resistance rates in the 95 isolates were 100% (95/95) for erythromycin, 68.42% (65/95) for tetracycline, and 53.68% (51/95) for streptomycin and ampicillin, respectively. The isolates were sensitive to polymyxin and sulfamethoxazole. Multi-drug resistance was seen in 70.53% (67/95) of the isolates. β-lactam-, aminoglycoside- and sulfonamide-encoding resistance genes were detected by PCR. The detection rate for *bla*_TEM_ and *sul3* was 100% (95/95), whereas *sul2* and *aaC4* had rates of 52.63 and 23.16%, respectively. These results indicate that some of the salmonellosis seen in Chinese breeding chicken farms may be caused by infection with *S. gallinarum-pullorum, S. enteritidis*, and *S. agona*. They also show that some *Salmonella* isolates have multi-drug resistance phenotypes and carry multi-drug resistance genes.

## Introduction

*Salmonella* is a food-borne disease-causing zoonotic pathogen (1), and its occurrence has frequently been reported in recent years (2, 3). More than 2,600 *Salmonella* serotypes have been identified and recorded (4). According to the World Health Organization (WHO), with nearly one-tenth of the world's population becoming infected each year and around 33 million deaths (http://www.who.int/mediacentre/factsheets/fs139/en/). Most cases occur in older adults and in immunocompromised people (5, 6), and it is estimated that in 2017 *Salmonella enterocolitis* was responsible for 95.1 million cases and killed 50,771 people (7–9). In addition to diarrhea, 535,000 cases of non-typhoidal *Salmonella* invasive disease have occurred, and an estimated 77,500 people have died (10). At present, antibiotic therapy remains the main prevention and treatment method for salmonellosis. However, the long-term and unreasonable use of antibiotics has caused *Salmonella* to develop widespread and strong resistance to these agents (11). Drug resistant bacteria can also spread to humans, leading to public health problems (12).

Various molecular typing techniques have been widely used in the field of microbiology to track the origins of pathogenic bacteria (13), the most widely of which used are multilocus sequence typing (MLST) and pulsed field gel electrophoresis (PFGE). MLST is fast and convenient, producing reliable high resolution data, and easy real-time internet sharing (14). PFGE is recognized by laboratories around the world for its high resolution and repeatability. But PFGE does not have a strict and uniform international naming standard, making it difficult to achieve data sharing with it. In recent years, a large number of epidemiological surveys on Salmonella have emerged in China. However, researchers were more inclined to conduct their epidemiological *Salmonella* surveys on commercial broiler sales chains where sick birds appeared than on healthier chains (15–17). Therefore, data from epidemiological surveys on *Salmonella* in domestic poultry farms, especially chicken breeder flocks, are incomplete.

Here, we collected 3,508 samples from 17 poultry breeding farms in nine Chinese provinces in 2019. We investigated the drug-resistance genes, drug-resistance phenotypes, and genetic relationships among the *Salmonella* isolates we collected. We comprehensively and systematically studied the epidemiological characteristics of *Salmonella*, with the aim of improving the molecular typing network database in China and providing relevant data with which to support the prevention and control of *Salmonella* in China.

## Materials and Methods

### Sample Collection

**Figure 1:**
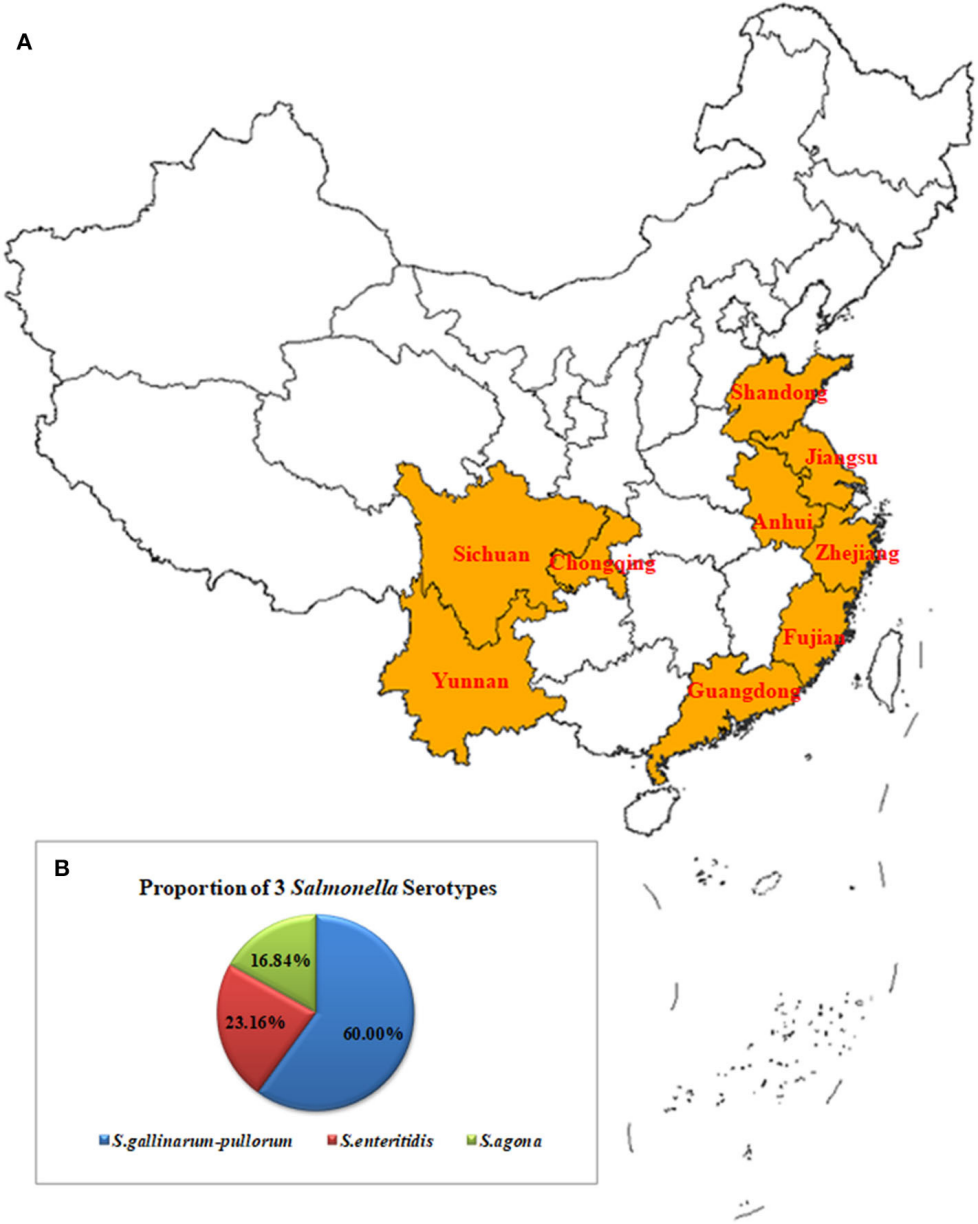
Geographical Map of the Chinese Sampling Sites and Proportions of the Serotypes for the *Salmonella* Isolates **(A)**. A total of 3,208 samples were collected from 14 poultry breeding farms in Jiangsu, Zhejiang, Anhui, Fujian, Guangdong, Yunnan, Chongqing, and Sichuan Provinces, China. They included 1,400 cloacal swab samples, 210 feed samples, 1,388 embryo samples, and 210 water samples. An additional 300 chicken embryo samples were obtained from three chicken breeding farms in Shandong Province, China **(B)**. The 95 isolates included 57 strains of *S. gallinarum-pullorum* (60.00%, 57/95), 22 strains of *S. enteritidis* (23.16%, 22/95), and 16 strains of *S. agona* (16.84 %, 16/95).

From May to July 2019, 3,208 samples were collected from 14 poultry breeding farms in Jiangsu, Anhui, Zhejiang, Fujian, Guangdong, Yunnan, Chongqing, and Sichuan Province, China. They included 1,400 cloacal swab samples, 210 animal feed samples, 1,388 embryo samples and 210 water samples. An additional 300 chicken embryo samples were obtained from three chicken breeding farms in Shandong Province, China (Figure 1A).

### Isolating and Serotyping *Salmonella*

Buffered peptone water (4.5 mL) (BPW, Haibo Biotechnology, Qingdao, China) was added to each sample (0.5 g) for pre-enrichment, according to a previously described method (18). After incubation at 37°C for 8–12 h, 0.5 mL of each pre-enriched culture was incubated in 4.5 ml of Tetrathionate Broth Buffer (TTB, Haibo Biotechnology) at 37°C for 24 h. After selective enrichment, one loopful of each broth culture was streaked onto xylose lysine tergitol 4 (XLT4, Haibo Biotechnology) agar and the plates were incubated at 37°C for 24–36 h. The presumptive *Salmonella* colonies were identified by polymerase chain reaction (PCR) assays using *invA* primers (19). The *invA* gene has a conserved sequence in *Salmonella* species and can therefore be used to detect and validate *Salmonella* strains (20). Here, *invA* primers (19) (F: 5′-ACAGTGCTCGTTTACGACCTGAAT-3′, R: 5′-AGACGACTGGTACTGATCGATAAT-3′) were used for PCR. The PCR cycling conditions were as follows: 1 denaturation cycle at 95°C for 5 min, 30 cycles of denaturation at 95°C for 30 s, followed by annealing at 56°C for 30 s and elongation at 72°C for 1 min, and a final 10 min elongation cycle at 72°C. Positive colonies were subsequently confirmed using a microbial mass spectrometer (IVD MALDI Biotyper, Bruker Bremen, Germany).

### *Salmonella* Serotyping

After being resuscitated on XLT4 medium, all the *Salmonella* isolates were serotyped according to the Kauffmann-White scheme by slide agglutination with O and H antigens (Tianrun Bio-Pharmaceutical, Ningbo, China). Normal saline was used as a negative control, and plate agglutination reactions were considered *Salmonella* positive. The *Salmonella* strains were PCR-identified using DNA as the template and *Ipaj* (F: 5′-TACCTGTCTGCTGCCGTGA-3′, R: 5′-ACCCTGCAAACCTGAAATC-3′) (21) as primers for detecting *S. gallinarum-pullorum*.

### Antimicrobial Susceptibility Testing

The Kirby-Bauer disk diffusion method, as described by the Clinical and Laboratory Standards Institute (CLSI), was used to test the susceptibility of the *Salmonella* isolates to 14 commonly used antibiotics, including polymyxin B (PB, 300 IU), sulfamethoxazole (SXT, 25 μg), florfenicol (30 μg), ofloxacin (5 μg), cefoxitin (30 μg), doxycycline (30 μg), amoxicillin (20 μg), tetracycline (TE, 30 μg), streptomycin (STR, 10 μg), gentamycin (10 μg), erythromycin (EM, 15 μg), ceftazidime (CAZ, 30 μg), ampicillin (AM, 10 μg), and gatifloxacin (5 μg). The results were interpreted according to the standard guidelines from the CLSI (22). *Salmonella* isolates that were resistant to more than three antimicrobial classes were defined as multidrug-resistant (MDR) isolates.

### Detecting Drug-Resistance Genes

Bacterial DNA was extracted from the samples using a Bacterial Genome Kit (Bioteke, Beijing, China), according to the manufacturer's instructions. The β-lactam genes (*bla*_*TEM*_*, bla*_*SHV*_*, bla*_*PSE*_*, bla*_*CTX*−*M*_, and *bla*_*OXA*_) and other genes associated with resistance to aminoglycosides (*aaC1, aaC3, and aaC4*), quinolones (*qnrB, qnrC, qnrD, qnrS*, and *aac*(6′)*-Ib-cr*), tetracyclines (*tetA, tetB*), and sulfonamides (*sul1, sul2*, and *sul3*) were PCR-detected. The relevant primers are listed in Table 1.

**Table 1:** Primers used to detect antimicrobial-resistance genes.

**Resistance gene category**	**Resistance gene**	**Primer sequence**	**References**
β-lactams	*bla_*TEM*_*	F: 5′- ATAAAATTCTTGAAGACGAAA – 3′	(23)
		R: 5′- GACAGTTACCAATGCTTAATC – 3′	
	*bla_*SHV*_*	F: 5′- TTATCTCCCTGTTAGCCACC – 3′	(23)
		R: 5′- GATTTGCTGATTTCGCTCGG – 3′	
	*bla_*PSE*_*	F: 5′- TAGGTGTTTCCGTTCTTG-3′	(24)
		R: 5′- TCATTTCGCTCTTCCATT-3′	
	*bla_*CTX*−*M*_*	F: 5′- CGCTTTGCGATGTGCAG-3′	(23)
		R: 5′- ACCGCGATATCGTTGGT-3′	
	*bla_*OXA*_*	F: 5′- TCAACTTTCAAGATCGCA-3′	(23)
		R: 5′- GTGTGTTTAGAATGGTGA-3′	
Quinolone	*qnrB*	F: 5′- GATCGTGAAAGCCAGAAAGG-3′	(23)
		R: 5′- ACGATGCCTGGTAGTTGTCC-3′	
	*qnrC*	F: 5′- GGTTGTACATTTATTGAATC-3′	(23)
		R: 5′- TCCACTTTACGAGGTTCT −3′	
	*qnrD*	F: 5′- AGATCAATTTACGGGGAATA-3′	(23)
		R: 5′- AACAAGCTGAAGCGCCTG – 3′	
	*qnrS*	F: 5′- ACGACATTCGTCAACTGCAA-3′	(23)
		R: 5′- TAAATTGGCACCCTGTAGGC-3′	
	*aac(6′)-Ib-cr*	F: 5′- TTGCGATGCTCTATGAGTGGCTA – 3′	(23)
		R: 5′- CTCGAATGCCTGGCGTGTTT – 3′	
Aminoglycosides	*aaC1*	F: ACCTACTCCCAACATCAGCC-3′	(25)
		R: ATATAGATCTCACTACGCGC-3′	
	*aaC3*	F: CACAAGAACGTGGTCCGCTA-3′	(25)
		R: AACAGGTAAGCATCCGCATC-3′	
	*aaC4*	F: CTTCAGGATGGCAAGTTGGT-3′	(25)
		R: TCATCTCGTTCTCCGCTCAT-3′	
Tetracycline	*tetA*	F: 5′- GCGCCTTTCCTTTGGGTTCT-3′	(25)
		R: 5′- CCACCCGTTCCACGTTGTTA-3′	
	*tetB*	F: 5′- CATTAATAGGCGCATCGCTG-3′	(25)
		R: 5′- TGAAGGTCATCGATAGCAGG-3′	
Sulfonamides	*sul1*	F: 5′- CTTCGATGAGAGCCGGCGGC-3′	(26)
		R: 5′- GCAAGGCGGAAACCCGCGCC-3′	
	*sul2*	F: 5′- GCGCTCAAGGCAGATGGCATT-3′	(26)
		R: 5′- GCGTTTGATACCGGCACCCGT-3′	
	*sul3*	F: 5′- AGATGTGATTGATTTGGGAGC-3′	(27)
		R: 5′- TAGTTGTTTCTGGATTAGAGCCT-3′	

### Detecting Class I Integrons

Class I integron-targeting primers were designed based on previously described sequences (28). After DNA gel extraction kit (Vazyme Biotech, Nanjing, China) purification, the class I integron-positive DNA samples were sequenced (Sangon Biotech, Shanghai, China). The DNA sequences obtained were compared with those in GenBank (https://www.ncbi.nlm.nih.gov/genbank/) using the Basic Local Alignment Search Tool to determine the gene cassettes within the variable region of the class I integrons.

### MLST

Seven housekeeping genes (*aroC, dnaN, hemD, hisD, purE, sucA*, and *thrA*) were used to characterize the *Salmonella* isolates using MLST (http://mlst.warwick.ac.uk/mlst/). The protocols from the MLST homepage were used, including the PCR conditions and primer sequence information (http://mlst.warwick.ac.uk/mlst/dbs/Senterica). PCR products were purified and sequenced (Sangon Biotech). The nucleic acid sequences corresponding to the allele values of the seven pairs of *Salmonella* housekeeping genes were downloaded from Pubmlst (https://pubmlst.org/), and BioEdit v7.0.9 software was used to construct a local gene bank for them. Genes from the gene library were aligned to obtain the allele values of the isolated strains and their corresponding ST types. The sequence types were assigned according to the MLST online scheme (http://mlst.warwick.ac.uk/mlst/dbs/Senterica), and the results of the eBURST map were constructed by Phyloviz (https://online.phyloviz.net/index).

## Results

### Isolation and *Salmonella* Serotyping

As described in the methods section, samples of the diseased materials were subjected to two-step enrichment using BPW and TTB, and a single colony was obtained by a three-line method on XLT4 medium. Colorless, translucent small colonies were observed on the plates, and some had smooth black cores and very narrow transparent bands around them. DNA was extracted from the isolated bacterial colonies, and PCR was performed with *invA* primers for *Salmonella* detection. After the PCR detection and the microbiological mass spectrometer verification, 126 *Salmonella strains were isolated, with the detection rate of* 3.59% (126/3508; Table 2). The isolating rates among different sample source were shown as: 6.27% (104/1688) in chicken embryo samples, 2.86% (6/210) in water samples, 1.14% (16/1400) in cloaca swabs, 0% (0/210) in feed samples. *Salmonella* was not detected in samples from Jiangsu and Sichuan provience. Among all the isolates, 54 sratins were recovered from samples of Shandong province, with the highest isolation rate of 18% (54/300), while a total of 72 *Salmonella* isolates were isolated from the other 6 provinces. In this study, a total of 95 strains, consisted of 23 randomly selected Shandong isolates and the aforementioned 72 isolates, were collected for subsequent tests.

**Table 2:** Isolation rate and distribution of chicken *Salmonella* isolates.

**Sampling area**	**Source**				**Isolation rate**
	**Feed**	**Water**	**Cloaca swabs**	**Embryo^a^**	
Jiangsu	0 (0/30)^b^	0 (0/30)	0 (0/200)	0 (0/200)	0% (0/460)
Zhejiang	0 (0/30)	6.67% (2/30)	7.00% (14/200)	4.00% (8/200)	5.22% (24/460)
Anhui	0 (0/30)	10% (3/30)	0.50% (1/200)	2.00% (4/200)	1.74% (8/460)
Fujian	0 (0/30)	0 (0/30)	0 (0/200)	13.00% (26/200)	5.65% (26/460)
Guangdong	0 (0/30)	0 (0/30)	0.50% (1/200)	4.00% (8/200)	1.96% (9/460)
Yunnan	0 (0/30)	3.33% (1/30)	0 (0/200)	1.00% (2/200)	0.65% (3/460)
Chongqing	0 (0/15)	0 (0/15)	0 (0/100)	2.00% (2/100)	0.87% (2/230)
Sichuan	0 (0/15)	0 (0/15)	0 (0/100)	0 (0/88)	0% (0/218)
Shandong	-	-	-	18.00% (54/300)	18.00% (54/300)^c^
Total	0 (0/210)	2.86% (6/210)	1.14% (16/1400)	6.27% (104/1688)	3.59% (126/3508)

a *All chicken embryos were dead embryos collected randomly from the hatchery that fail to hatch*.

b *Numbers in parentheses are positive/total*.

c *We randomly selected 23 strains from 54 strains for in-depth study*.

### *Salmonella* Serotyping Results

The Ningbo Tianrun Serum Diagnostic Kit test results showed that the O antigens of the 95 *Salmonella* strains were O9, O12 (79/95) and O4, O12 (16/95). The results from the semi-solid puncture tests showed that 38/95 isolates had flagella and 57 had no flagella. The serum diagnostic kit results showed that the H antigens from the 38 isolates were Hg, m (22/38) and Hs (16/38). The kit's results when combined with the PCR results showed that the 95 isolates included 57 *S. gallinarum-pullorum* strains (60.00%, 57/95), 22 *S. enteritidis* strains (23.16%, 22/95), and 16 *S. agona* strains (16.84 %, 16/95) (Figure 1B).

### Antibiotic Resistance and MDR Profiles

**Figure 2:**
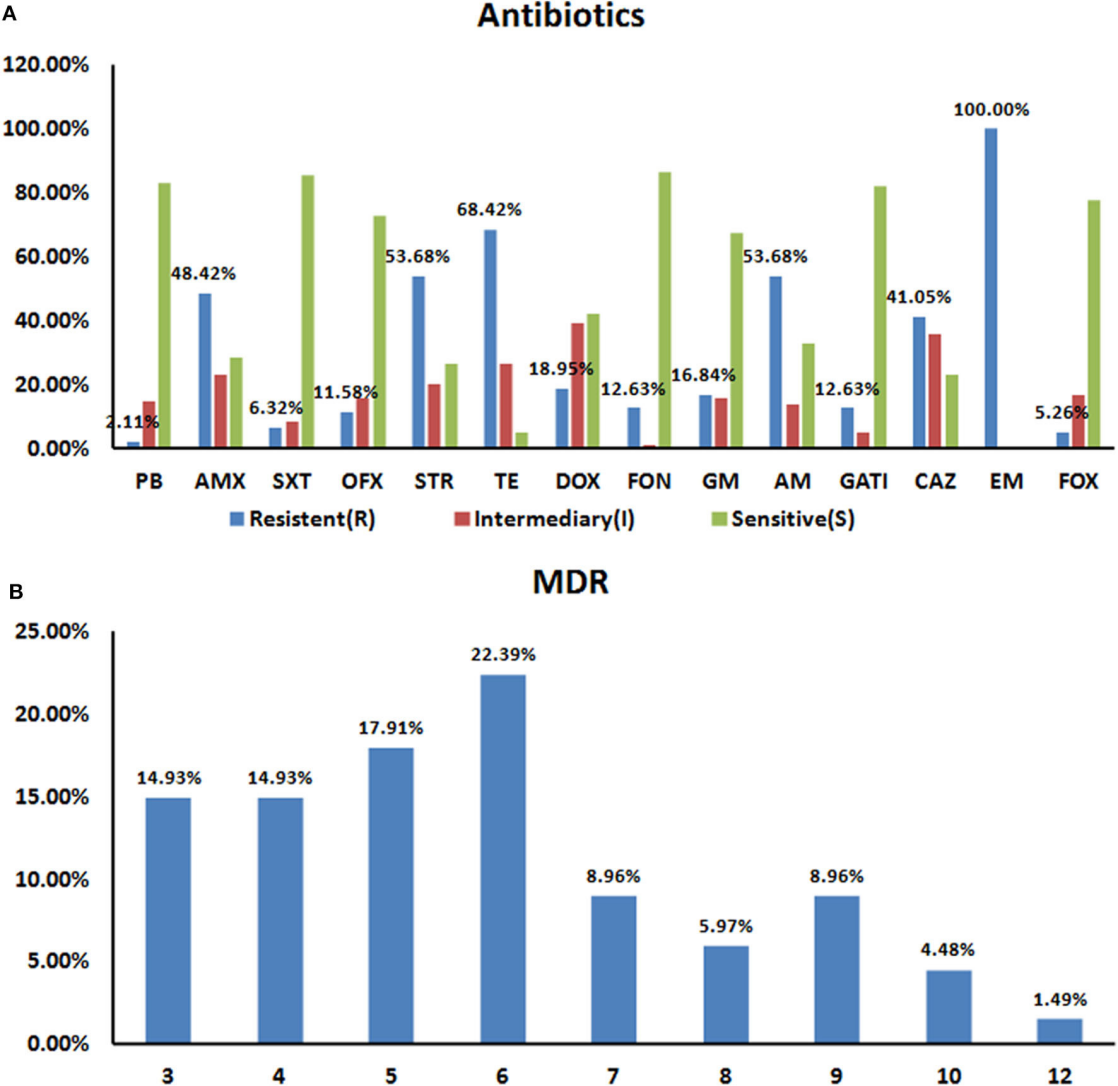
Resistance Characteristics of the 95 *Salmonella* Strains to 14 Antibiotics **(A)**. Rates of antibiotic resistance amongst *Salmonella* isolates. The drug resistance rates for all 95 isolates were 100% for EM (95/95), 68.42% (65/95) for TE, and 53.68% (51/95) for STR and AM, respectively. All 95 strains showed polymyxin and sulfamethoxazole sensitivity **(B)**. Prevalence of multidrug resistance amongst 95 *Salmonella* isolates. 70.53% (67/95) of the tested *Salmonella* isolates were resistant to at least three antibiotics. About 36.8% (35/95) strains resistant to 6–12 drugs.

The KB method was used to count the diameters of the inhibition zones and to determine growth inhibition to 14 common antibacterial drugs in the 95 *Salmonella* strains according to the 2018 edition of the CLSI (22) standards. The resistance rates in the 95 isolates were 100% (95/95) for erythromycin, 68.42% (65/95) for tetracycline, and 53.68% (51/95) for STR and AM, respectively. The AM resistance rate for the 95 isolates was 53.68% (51/95), and they were all sensitive to PB and SXT (Figure 2A).

**Figure 3:**
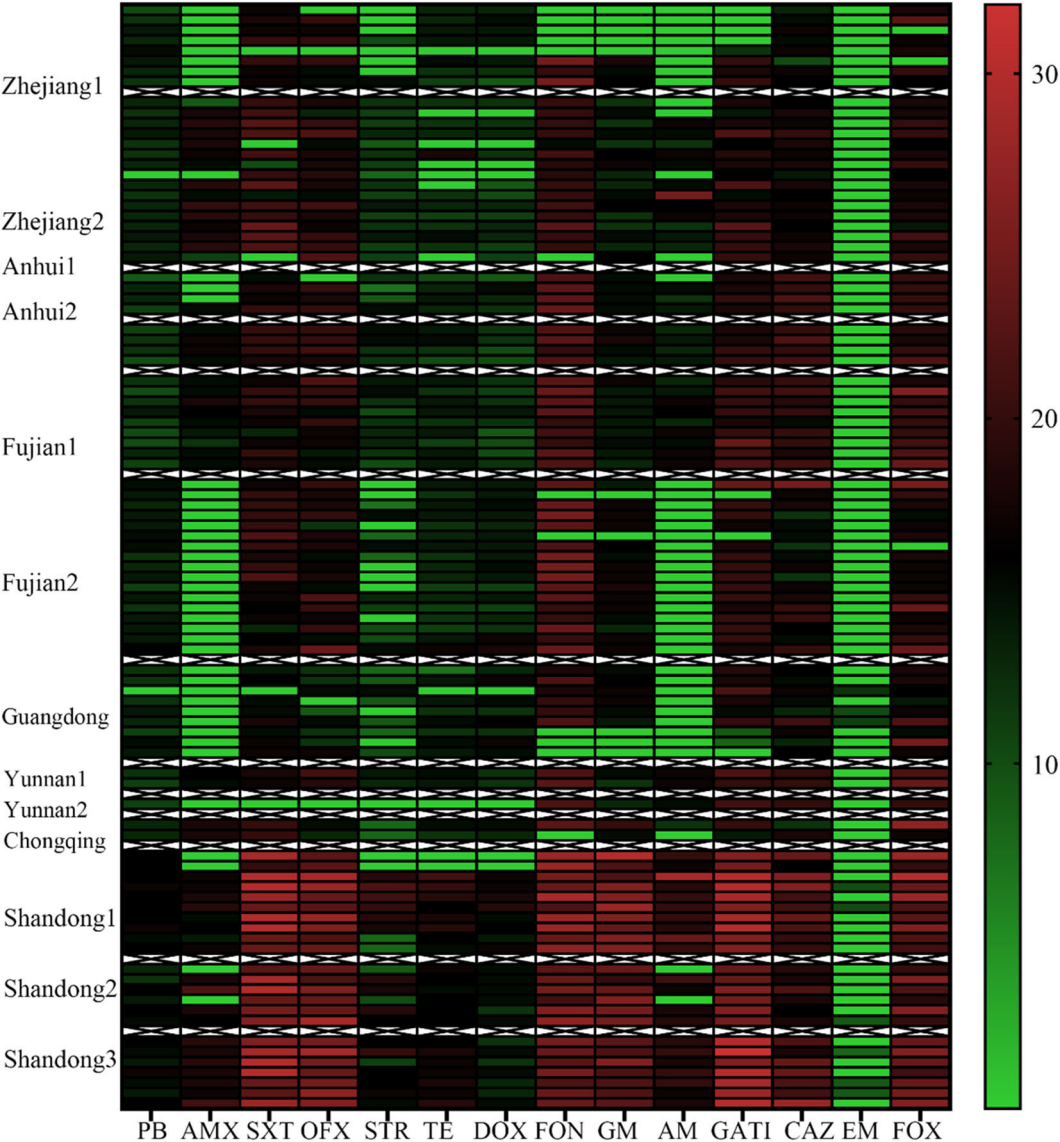
Antibiotic Resistance Heat Map for the 95 *Salmonella* Strains. Heat map showing the antibiotic-resistance distribution for the 95 *Salmonella* strains isolated from samples from broiler chicken farms in nine provinces to 14 antibiotic types. The color scale of the individual cells represents the resistance rate, as a percentage. PB, polymyxin B; AMX, amoxicillin; SXT, sulfamethoxazole; OFX, ofloxacin; STR, streptomycin; TE, Tetracycline; DOX, doxycycline; FON, florfenicol; GM, Gentamycin; AM, ampicillin; GATI, Gatifloxacin; CAZ, Ceftazidime; EM, erythromycin; and FOX, cefoxitin.

We next statistically analyzed the drug resistance spectrum and multiple drug resistance in the isolated bacteria (Figure 2B). The MDR statistics showed that 70.53% (67/95) of the strains were resistant to three or more drug types, indicating that drug resistance in the isolated bacteria is prevalent with 38 different resistance patterns identified (Table 3). The drug-resistance spectrum was broad, and the most common pattern in seven of the isolates involved AMX-STR-TE-AM-CAZ-EM. The serotypes and resistance patterns were not significantly correlated. According to the resistance profile heat map (Figure 3), drug-resistant *Salmonella* isolated from the three chicken farms in Shandong Province had the highest drug-resistance rates.

**Table 3:** Antimicrobial resistance patterns of the 95 *Salmonella* isolates.

**No. of drugs**	**Antimicrobial resistance patterns**	**Isolate no**.
12	AMX-SXT-OFX-STR-TE-DOX-FON-GM-AM-GATI-CAZ-EM	1
10	AMX-OFX-STR-TE-FON-GM-AM-GATI-CAZ-EM	2
	AMX-STR-TE-FON-GM-AM-GATI-CAZ-EM-FOX	1
9	AMX-STR-TE-FON-GM-AM-GATI-CAZ-EM	5
	AMX-OFX-STR-FON-GM-AM-GATI-CAZ-EM	1
8	PB-AMX-STR-TE-DOX-AM-CAZ-EM	2
	AMX-SXT-STR-TE-FON-AM-CAZ-EM	1
	AMX-OFX-STR-TE-AM-CAZ-EM-FOX	1
7	SXT-STR-TE-DOX-GM-AM-EM	1
	AMX-OFX-STR-TE-AM-CAZ-EM	4
	AMX-SXT-OEX-STR-TE-DOX-EM	1
6	AMX-STR-AM-CAZ-EM-FOX	2
	AMX-STR-TE-AM-CAZ-EM	7
	AMX-TE-DOX-AM-CAZ-EM	1
	AMX-TE-GM-AM-CAZ-EM	1
	STR-TE-DOX-AM-GATI-EM	1
	TE-GM-AM-CAZ-EM-FOX	1
	AMX-OFX-TE-DOX-AM-EM	1
	STR-TE-FON-AM-GATI-EM	1
5	STR-TE–GM-CAZ-EM	2
	SXT-STR-TE-DOX-EM	1
	STR-TE-DOX-CAZ-EM	1
	AMX-STR-TE-AM-EM	5
	AMX-TE-AM-CAZ-EM	1
	AMX-STR-TE-DOX-EM	2
4	STR-TE-AM-EM	1
	AMX-STR-TE-EM	1
	TE-DOX-AM-EM	1
	TE-AM-CAZ-EM	1
	AMX-TE-DOX-EM	1
	AMX-TE-AM-EM	1
	AMX-STR-AM-EM	3
	STR-AM-CAZ-EM	1
3	TE-DOX-EM	5
	TE-CAZ-EM	1
	TE-AM-EM	2
	STR-TE-EM	1
	AMX-AM-EM	1

### Prevalence of Antibiotic Resistance Genes

**Figure 4:**
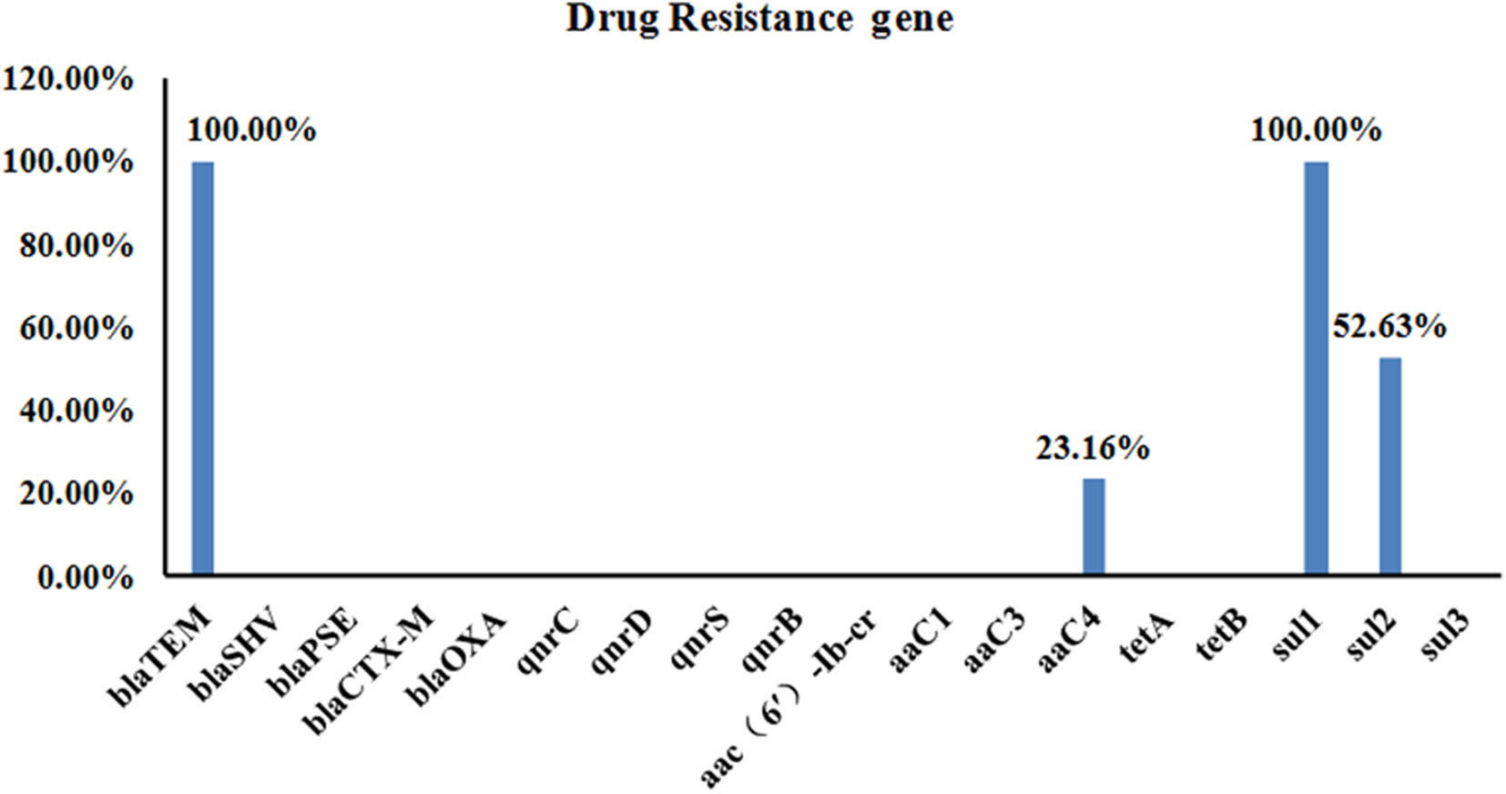
Carriage of 18 Drug Resistance Genes in the 95 *Salmonella* Strains. A total of four different drug resistance genes were detected in the DNA from the isolates, including those encoding β-lactams, aminoglycosides, and sulfonamides. The highest detection rate was for *bla*_*TEM*_ and *sul3* (both were 100%, 95/95), followed by *sul2* and *aaC4* with detection rates of 52.63 and 23.16%, respectively.

Using the DNA from each isolate as the template, PCR was used to detect the carriage status of the isolates to 18 drug resistance genes. Four different drug resistance genes were detected in DNA from the isolates, including those encoding β-lactams, aminoglycosides, and sulfonamides. The highest detection rates were seen for *bla*_*TEM*_ and *sul3* (100%, 95/95), followed by *sul2* and *aaC4* (52.63 and 23.16%, respectively) (Figure 4).

### Detection of Class I Integrons

PCR testing detected no class I integrants in the DNA from the isolates; therefore none of the strains from this study carried class I integrants.

### MLST

**Figure 5:**
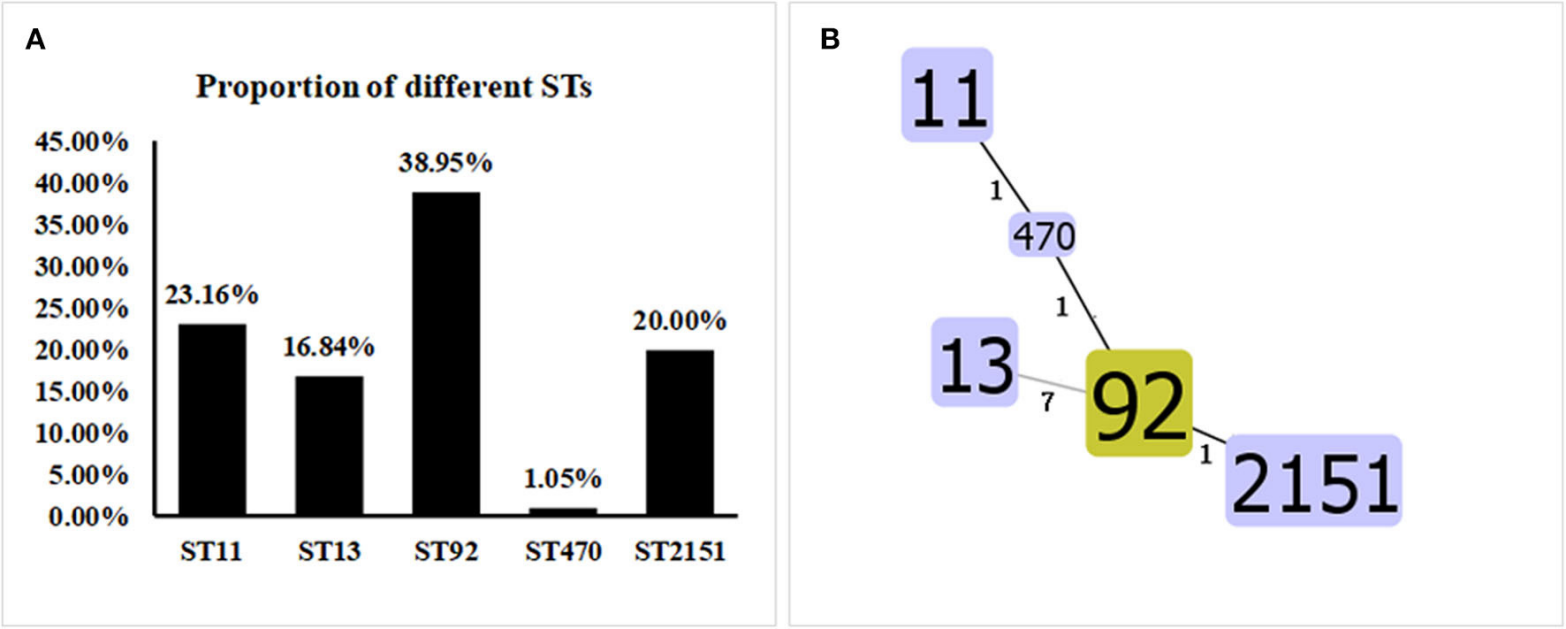
Proportions and Allelic Map of the Five STs in the 95 *Salmonella* Isolates. **(A)** MLST classification showed that the 95 *Salmonella* strains fell into the following five STs: ST92 (37/95, 38.95%), ST11 (22/95, 23.16%), ST2151 (19/95, 20.00%), ST13 (16/95, 16.84%), and ST470 (1/95, 1.05%). **(B)** Apart from ST13, the other four STs shared close genetic relationships. The genetic direction was found to be ST11-ST470-ST92-ST2151, and ST11 is the core sequence type.

MLST classification showed that the 95 *Salmonella* strains fell into five ST types: ST92 (37/95, 38.95%), ST11 (22/95, 23.16%), ST2151 (19/95, 20.00%), ST13 (16/95, 16.84%), and ST470 (1/95, 1.05%) (Figure 5A). The results of the eBURST map showed that isolate ST11 was a cloned progenitor, isolates ST92 and ST2151 were subcloned groups from isolate ST11. Apart from ST13, the other four STs have close genetic relationships. The genetic direction was determined to be ST11-ST470-ST92-ST2151 (Figure 5B), with ST11 being the core sequence type.

## Discussion

An important transmission route for *Salmonella* is vertical transmission. Therefore, chicken breeder farms are required to actively prevent, control, and work toward eliminating *Salmonella* infections on them. Once breeder chickens become infected with *Salmonella*, a “magnification effect” can occur as the generations expand. China has listed Salmonellosis as a priority disease control species in the National Medium and Long-Term Animal Disease Prevention and Control Plan (2012–2020) (http://www.gov.cn/zwgk/2012-05/25/content_2145581.htm), which requires all chicken farms across the country to meet the standards required to eliminate chicken salmonellosis by 2020. Hence, investigating *salmonella* epidemics on breeding farms is particularly important.

To investigate the prevalence and characteristics of *Salmonella* on Chinese poultry breeding farms requires information about drug-resistance genes, and the phenotypic characteristics of the isolates collected from them and their relationships. In this study, from May to July 2019, 3,508 samples from breeding farms were collected, including 1,400 cloaca swabs, 210 feed samples, 1,688 chicken embryo samples, and 210 water samples. The samples came from 14 breeder farms in eight provinces (Jiangsu, Anhui, Zhejiang, Fujian, Guangdong, Yunnan, Chongqing, and Sichuan) and three breeder flocks in Shandong Province. We detected 126 *Salmonella* strains in the 3,508 samples, a positivity rate of 3.59%. The positive isolation rate was very low, which may reflect the fact that the efforts made to prevent and control animal diseases in China are transitioning from effective control to gradual purification and elimination. In this investigation, especially in several breeder flocks in Jiangsu and Sichuan, *Salmonella* elimination has been done well. In contrast, the three local breed chicken breeder flocks in Shandong were more seriously contaminated with *salmonella*. It indicated that more attention should be paid on *salmonella* purification in local chicken breeds.

From the 1990s onwards, pullorum disease has seriously threatened the poultry industry (29). *S. pullorum* infection is still threatening the poultry farming industry and related industries in developing countries including China (30), resulting in huge economic losses in these countries. Among the three serotypes identified in this study, *S. gallinarum-pullorum* (57/95, 60.00%) still dominates, with *S. enteritidis* (22/95, 23.16%) and *S. agona* (16/95, 16.84%) ranking second and third. In contrast, *S. enteritidis, S. Indiana*, and *S. typhimurium* are more common in the Chinese chicken retail industry.

After serotype identification, we used molecular typing to further analyze the genetic relationships among the isolates. Various molecular typing techniques are widely used in the microbiology field to track the origin of pathogenic bacteria (13), from which the most commonly used techniques are MLST and PFGE. The high resolution and repeatability of PFGE has been recognized by laboratories around the world, but PFGE lacks a strict and uniform international naming standard, so it is difficult to achieve data sharing with this technique. With its convenience, high resolution, reliable data, and easy real-time internet sharing, MLST is widely used (14). In this study, the MLST typing results showed that the 95 *Salmonella* strains we isolated fell into five ST types (ST92, ST11, ST2151, ST13, and ST47) (1/95, 1.05%). The following correlations between STs and *Salmonella* serovars were founded: ST13 with *S. agona*, ST11 with *S. Enteritidis*, and ST92, *ST2151*, and ST470 with *S. gallinarum-pullorum*. Among them, ST92 (37/95, 38.95%) was the most common, a finding consistent with the results of a study on *S. enteritidis* in China from 2011 to 2016 (30), thereby confirming that the current *Salmonella* type still dominates. In addition to ST13, the *Salmonella* isolates displayed four other close ST kinships, with the genetic direction being ST11-ST470-ST92-ST2151.

In this study, β-lactams, quinolones, aminoglycosides, tetracyclines, and sulfonamides were tested on the 95 isolates. Among them, β-lactam-, aminoglycoside-, and sulfonamide-resistance genes were detected by PCR. Four resistance genes were detected, among which the detection rate for the β-lactam *bla*_*TEM*_resistance gene and the *sul3* sulfonamide resistance gene was 100% (95/95) for both, whereas those of *sul2* and *aaC4* were 52.63 and 23.16%, respectively. Quinolone resistance genes and tetracycline resistance genes were not detected. This result is consistent with the results of a study (31) on sulfonamide-resistance genes in *Pasteurella multocida*, where the detection rate for *sul2* (and *sul1*) exceeded 97%. Furthermore, Zhu et al. (32) identified sulfonamide-resistance genes (*sul1, sul2*, and *sul3*) in 89 (97.8%) Salmonella isolates. These findings indicate that sulfonamide-resistance genes are relatively stable in the environment. Extended-spectrum β-lactamase (ESBL)-producing *Salmonella* is a significant clinical and food safety concern worldwide (33). *Salmonella* isolates that harbor ESBL-encoding genes are able to hydrolyze penicillin as well as most of the first, second, and third generation cephalosporins, and even carbapenems (34, 35). In the present study, all the *Salmonella* isolates carried *bla*_*TEM*_, a β-lactam resistance gene, highlighting their potential pathogenesis and the risk posed by this pathogen to Chinese poultry breeding farms.

The Chinese Veterinary Pharmacopeia report (30) pointed out that the drugs tested in the present study, including EM, TE, SXT, and AM, have been widely used in poultry production in China. The resistance rate of these 95 isolates to AM, STR, TE, and EM ranged from 53.68 to 100%. The *Salmonella* isolates from each province in this article were resistant to at least three common antibacterial drugs, and the proportion of MDR strains was 70.53% (67/95), which is consistent with the results from previous surveys (15, 36). This result is consistent with the findings of previous reports from Iran and the United States (37, 38). It is worth noting that should strains with the same MDR genotypes as those identified in the *Salmonella* isolates be transferred to humans via chickens and their derivatives and with different degrees of resistance to 14 antibacterial drugs as was shown for our isolates, this would constitute a major public health threat.

A recent study indicated that integrins and multiple drug resistance genes on mobile plasmids may be responsible for the stable spread of MDR genes in *Salmonella* (39). Previous studies on class I integrants in *Salmonella* in Hubei, Shandong, and Shanxi (18, 39, 40) provinces have found that class I integrants displaying the various antibiotic resistance gene cassettes that are commonly found in *Salmonella* isolates are associated with provinces, markets, and storage conditions differ. Multiple antibiotic resistance in isolates carrying class I integrons is significantly higher than that seen in isolates lacking these integrons, and they usually show a corresponding antibiotic resistance spectrum to the antibiotic resistance gene cassette contained in their class I integrants. In the present study, the detection rate for class I integrants was 0 (0/95), and the influence of gene mutations and other factors relating to drug resistance in *Salmonella* was not investigated. This may be related to the stricter control measures of breeding poultry farms compared to commercial chicken farms, bacterial infectious diseases are better controlled, and the application of antibiotics is greatly reduced. At the same time, it should be noted that poultry farming often uses antibiotics, which may explain the resistance patterns we observed with the tested antibiotics. Therefore, reducing antibiotic use is particularly important for limiting the emergence of super multidrug resistant organisms so that good health in humans and animals can be maintained.

## Conclusions

The *Salmonella* detection rate for the 17 breeder farms in the Chinese provinces we surveyed was 3.59%. Five STs, ST92, ST11, ST2151, ST13, and ST470 were identified. Our results indicate that among the *Salmonella* prevalent in the poultry breeder farms in the various provinces, *S. gallinarum, S. enteritidis*, and *S. agona* still occupy the main positions. All of the *Salmonella* isolates carried *bla*_*TEM*_ and *sul3*, and 64.21% were MDR strains, suggesting that antibiotic use in the poultry breeder farms still requires attention. The correlation between the serotypes and the molecular subtypes of the *Salmonella* isolates suggests that the results of a comprehensive analysis of different methods may provide more useful epidemiological information related to this infection. Our findings will update current knowledge on the prevalence of antibiotic resistance on poultry breeding poultry farms in China and provide information and technical support toward the long-term goal of eradicating salmonellosis.

## Data Availability Statement

The raw data supporting the conclusions of this article will be made available by the authors, without undue reservation.

## Author Contributions

SS, FW, and XG conceived and designed the experiments and analyzed the data. YanS, FZ, LZ, and FW performed the experiments and contributed the reagents. YL performed data validation. FW and YanyS wrote the manuscript. All authors have read and approved the final manuscript.

## Conflict of Interest

The authors declare that the research was conducted in the absence of any commercial or financial relationships that could be construed as a potential conflict of interest.
